# Bio-Aerogels as Materials for Active Food Packaging: Emerging Trends in Food Preservation

**DOI:** 10.3390/gels11090756

**Published:** 2025-09-19

**Authors:** Yuliza G. Morales-Herrejón, Jorge Vargas-Almaraz, Adolfo Castañeda-Salazar, Sandra Mendoza

**Affiliations:** Research and Graduate Studies in Food Science, School of Chemistry, Universidad Autónoma de Querétaro, Santiago de Querétaro 76010, Querétaro, Mexico; yulizamorales89@gmail.com (Y.G.M.-H.); jvargas10@alumnos.uaq.mx (J.V.-A.)

**Keywords:** aerogels, active packaging, food preservation, sustainability, functional packaging, packaged materials

## Abstract

Active food packaging is an innovative strategy to improve preservation and extend the shelf life of food products. In this context, aerogels, extremely lightweight and porous materials, have gained popularity for packaging development due to their ability to integrate active properties into their structure that enhance moisture control, controlled drug release, and barrier properties. This review explores emerging trends in the use of aerogels for active food packaging, focusing on the physical and functional properties of aerogels, their current applications in the food industry, and the challenges associated with their large-scale adoption. In addition, aerogels are compared to conventional packaging materials, highlighting their advantages in terms of sustainability and performance. Despite the numerous benefits and great potential of aerogels in the food industry, concerns related to cost, mechanical strength, and food safety persist. Recent developments in the production of aerogels and prospects for their use as an innovative material in packaging are addressed.

## 1. Introduction

Food packaging plays a crucial role in preserving both fresh and processed products by protecting them from external factors such as atmospheric exposure, microbial contamination, and mechanical damage. This protection is essential for maintaining food safety, integrity, freshness, and quality throughout its shelf life. Traditionally, plastic packaging has dominated the industry due to its versatility, low cost, and excellent barrier properties [1]. However, conventional plastics often fall short in preventing food spoilage, contributing to significant levels of food waste. This increases food loss and environmental pollution, particularly due to their lack of biodegradability and reliance on fossil fuels [2].

In response to growing environmental concerns, the food industry is transitioning towards more sustainable packaging alternatives. Biopolymers, derived from renewable and sustainable resources, have emerged as a promising solution, with a market valued at USD 17.54 billion in 2023 and projected to reach USD 38.69 billion by 2030, growing at a Compound Annual Growth Rate (CAGR) of 10.4% from 2024 to 2030. Furthermore, the global food packaging market was valued at USD 400.29 billion in 2024, with a projected CAGR of 5.9% from 2025 to 2030 [3].

The intensified demand for bio-based food packaging has resulted in the development of active packaging, which, compared with traditional food packaging, not only protects the food from the external environment but also contains active ingredients that control deterioration, oxidation, and flavor changes in the food. Active food packaging can release or absorb substances, maintaining the quality and the safety of food. Recently, active food packaging enhancement has been achieved by using micro- and nanostructured materials [1].

Among the most innovative structured materials under investigation are bio-based aerogels, lightweight, highly porous structures capable of performing active functions in food packaging. These materials can modulate water activity, absorb excess moisture, and enable the controlled release of antimicrobial or antioxidant agents, thereby prolonging the shelf life of food products. This review provides a comprehensive overview of the emerging role of bio-aerogels in active food packaging. We focus on their production methods, physical and functional properties, precursor materials, current limitations, and future perspectives for their implementation in sustainable food packaging systems [4].

## 2. Review Methodology

**Figure 1:**
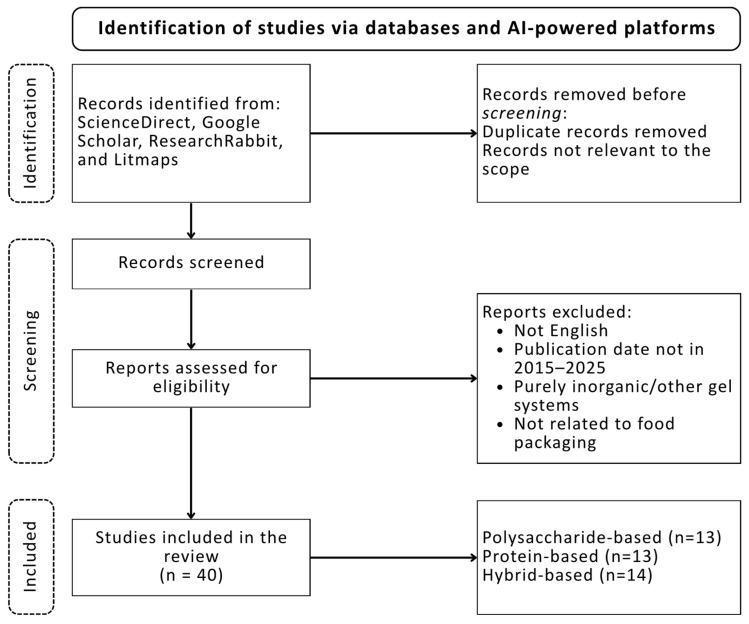
Flow chart of the systematic review. Caption: PRISMA flow chart of the systematic review, detailing the search databases, the number of articles selected, and the exclusion criteria.

**Figure 2:**
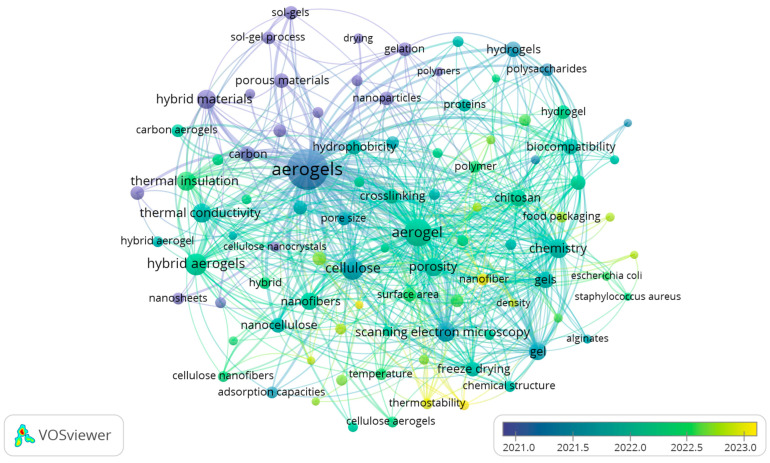
Overview of hot research topics on bio–aerogels. Figure created with VOSviewer 1.6.20.

The literature search and classification strategy followed the guidelines proposed by [5]. Two international databases, ScienceDirect and Google Scholar, were systematically screened for article titles, keywords, and abstracts using quaternary combinations of the term “aerogels” with “polysaccharide”, “protein”, “hybrid”, and “food packaging”, connected through the Boolean operator AND. Additionally, the term “bio-aerogels” was used independently to capture studies with a broader scope. The search was limited to publications from 2015 to 2025. The systematic review methodology is presented in the PRISMA flow chart (Figure 1). This chart shows the number of studies selected and ultimately included in the review according to the inclusion and exclusion criteria. The use of bio–aerogels in different areas as food packaging has been growing rapidly over the last years (Figure 2). Henceforth, the objective of this work is to review the state–of–art use of bio–aerogels and to provide a detailed overview of their application in food packaging.

## 3. Origin and Evolution of Aerogels

The development of aerogels began in 1931, when Steven Kistler synthesized the first material of this kind by replacing the liquid phase of a gelatinous substance with a gas through supercritical drying. This process involves increasing both temperature and pressure beyond the critical point of the fluid, thus avoiding liquid evaporation and preventing the collapse of the porous network due to capillary forces [6]. Kistler experimented with various precursor materials, including silica, tin oxide, and cellulose. Notably, cellulose-based aerogels were the first from organic sources.

Since then, the interest in fabricating and characterizing aerogels has increased considerably, driven by their unique structural properties and broad application potential. Despite this, the definition of what constitutes an aerogel continues to evolve, as their physicochemical characteristics vary significantly depending on the synthesis conditions, particularly the drying method used [7].

According to the International Union of Pure and Applied Chemistry (IUPAC), aerogels are defined as “non-fluid networks composed of interconnected colloidal particles dispersed in a gas phase (typically air)”. More broadly, they are considered solid materials with a highly porous three-dimensional structure and very low density, obtained by replacing the liquid phase of a gel with a gas. Their classification depends primarily on the drying technique employed:

- Aerogels, obtained via supercritical drying, generally exhibit a mesoporous structure, with pore diameters between 2 and 50 nm.
- Xerogels, formed through ambient pressure drying, tend to be microporous, with pores smaller than 2 nm.
- Cryogels, produced by freeze-drying, are typically macroporous, with pore sizes exceeding 50 nm.

Although only supercritically dried materials are strictly defined as aerogels, xerogels and cryogels are often considered as aerogels due to their similar textural characteristics [8,9].

The evolution of aerogel research has progressed along two main axes: the diversification of precursor materials and the expansion of potential applications. Initially dominated by inorganic compounds and synthetic polymers, recent developments have focused on natural polymers and hybrid materials to enhance functionality and sustainability. Concurrently, aerogels have expanded their utility into diverse fields, including environmental remediation, biomedicine, advanced materials science, and, more recently, food engineering [10]. This broadening of focus and innovation is illustrated in the bibliometric trends depicted in Figure 2.

## 4. Properties of Bio-Aerogels and Their Applications

Bio-aerogels are obtained from a hydrogel based on natural matrices such as cellulose and its derivatives, proteins and peptides, polysaccharides, and lipids, among others [1,8]. Its distinctive characteristics depend largely on the precursor material and include an ultra-low bulk density (0.0001–0.2 g/cm^3^), low thermal conductivity (around 0.015 W/m·K), high specific surface area (greater than 200 m^2^/g), and an open, highly porous structure (95% to 99%), predominantly in the mesoporous range (2–50 nm) [8,10,11,12].

**Figure 3:**
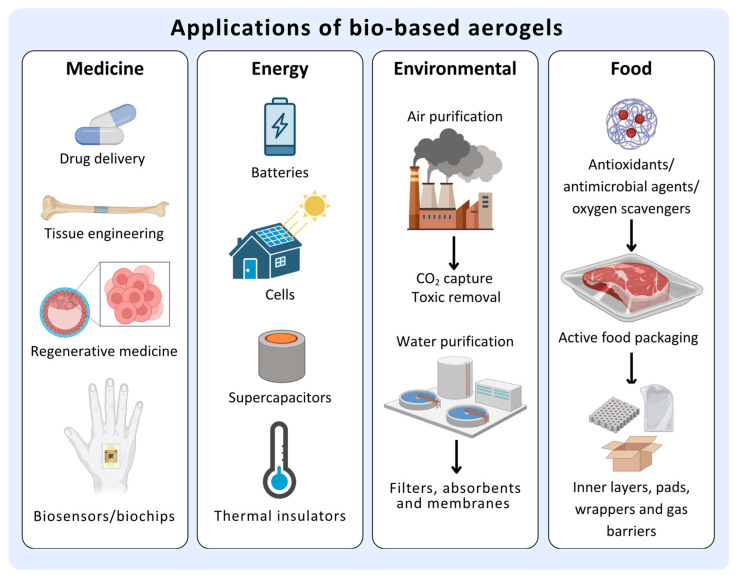
Applications of bio-aerogels in areas such as medicine, energy, environmental remediation and food industry. Figure created using information from [12] and designed with BioRender.com.

These attributes confer bio-aerogels with exceptional thermal insulation capabilities, in addition to high load and release capacity of compounds. Even though bio-based aerogels have a wide range of applications, including in medicine, energy sciences, environmental remediation, and electromagnetic interference attenuation, recently, they have also been incorporated into the food industry (Figure 3) [12].

The high biodegradability and biocompatibility of bio-aerogels have increased research into their use in food applications. In this context, bio-aerogels can be used for active packaging, for example, as inner antioxidant layers, absorbent pads, antimicrobial wrappers, gas barriers, and fat replacements, as well as in edible delivery systems [13,14,15,16,17].

The primary aim of active food packaging is to protect food products from environmental and mechanical stress factors, such as exposure to gases, moisture, light, and sudden temperature fluctuations, as well as microbial growth and contamination [16]. The selection of packaging materials depends on both their protective performance and their ability to fulfill other essential functions, such as containment, transport, product presentation, and delivering consumer information.

In this context, bio-aerogels demonstrate remarkable functional versatility, rendering them suitable for primary and secondary packaging. They are often combined with tertiary systems to facilitate transport and storage. Furthermore, their porous structure allows antimicrobial or antioxidant agents to be released, which effectively extends the shelf life of food products.

## 5. Bio-Aerogels Manufacturing Stages for Food Applications

**Figure 4:**
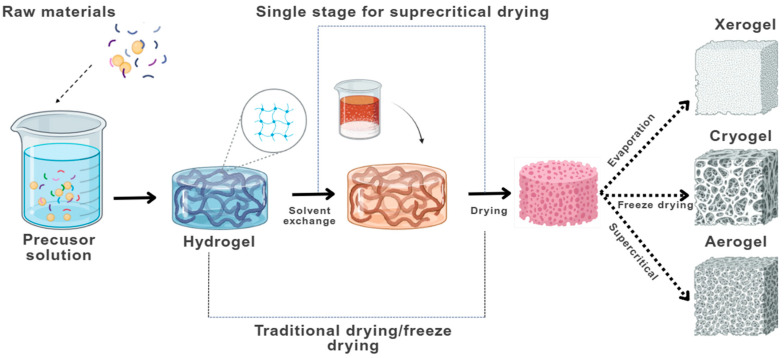
General procedure for obtaining bio-aerogels. Figure adapted from [25] and designed with BioRender.com.

The fabrication of bio-aerogels intended for food applications generally comprises three fundamental stages: (i) hydrogel formation, (ii) solvent exchange (when applicable), and (iii) drying, which finalizes the porous bio-aerogel structure [17]. A schematic overview of these stages is illustrated in Figure 4.

### 5.1. Hydrogel Formation

The initial step to produce aerogels involves the development of a three-dimensional hydrogel network, in which a liquid phase, commonly water, is entrapped within a polymeric or colloidal matrix. For food-related uses, this matrix typically consists of natural biopolymers such as proteins, polysaccharides, or their blends, including those obtained from agricultural by-products like bagasse, which is mainly composed of polysaccharides and proteins. Gelation can be triggered by various mechanisms, including thermal treatment, pH adjustment, ionic cross-linking, and addition of gelling agents, depending on the biopolymer and desired properties [8,18].

### 5.2. Solvent Exchange

In supercritical drying, a solvent exchange step is performed before drying, where water is replaced with a less polar solvent such as ethanol or acetone. This step reduces surface tension effects, thereby minimizing capillary forces during drying and helping to preserve the porous structure of the gel. The efficiency of this step can directly influence the textural integrity and performance of the final bio-aerogel [12].

### 5.3. Drying

The final stage of the process involves the removal of the liquid phase to yield a dry, highly porous material. The drying technique employed is of critical importance in defining the bio-aerogel’s microstructure, porosity, and functional properties. The most common methods for producing bio-aerogels are supercritical drying, freeze-drying, and ambient pressure drying. Each of these methods results in bio-aerogels with distinct physical characteristics and performance profiles.

a. Air drying

Air drying, also known as ambient pressure drying, consists of evaporating the solvent from the gel matrix at ambient conditions or in a convection oven at controlled temperatures. Although this method is economically attractive and technically simple, it often results in significant pore collapse, shrinkage, and crack formation due to the high capillary stress generated during solvent evaporation. These structural changes compromise the characteristic nanoporous architecture of bio-aerogels (or xerogels). Nonetheless, optimization of drying parameters such as temperature, humidity, and drying rate can partially improve the final properties of air-dried aerogels [19,20].

b. Freeze-drying

Freeze-drying involves the freezing of the hydrogel, generally from −50 to −80 °C, followed by sublimation of the solvent under reduced pressure. This method better preserves the porous structure than air drying, minimizing shrinkage and cracks. However, the formation of anisotropic pore architectures can occur, depending on the freezing direction and rate, which may affect the uniformity of the final bio-aerogel [17].

c. Supercritical drying

Supercritical drying is widely regarded as the most effective technique for preserving the original gel structure. In this method, the solvent is replaced with supercritical carbon dioxide (CO_2_), and the system is depressurized without crossing the liquid–gas phase boundary. This eliminates capillary stress, allowing for the retention of a highly porous and low-density structure with minimal shrinkage. Despite its advantages, supercritical drying is more expensive and technically demanding, limiting its widespread use in food applications [21,22].

However, the use of agro-industrial waste (biomass) presents several significant advantages that offset the high cost of supercritical drying in aerogel manufacturing. Biomass includes materials such as cellulose, hemicellulose, and proteins derived from plants, animals, and marine organisms, representing a renewable, abundant, and low-cost source of resources [12]. This vast availability contributes to the cost-effectiveness of bio-aerogels, even those obtained by supercritical drying [23]. A review by Wei et al. [12] details a sustainability analysis of biomass aerogels versus plastic-based aerogels and projects a future decrease in the costs of raw materials obtained from agro-industrial waste. Some examples of these wastes include old corrugated containers, bamboo residues, corn straw, and rice and oat husks, among others. Therefore, the use of agro-industrial waste contributes to minimizing the accumulation of waste and pollution caused by conventional plastic materials [1].

Several studies have compared the effects of these drying methods on protein-based aerogels. For instance, Plazzotta et al. [24] reported that whey protein bio-aerogels produced via supercritical drying exhibited a more homogeneous porous structure and reduced particle aggregation compared to those obtained through freeze-drying. The bulk densities of aerogels produced by supercritical drying and freeze-drying were reported as 0.021 g/cm^3^ and 0.070 g/cm^3^, respectively, highlighting the impact of drying technique on final material properties. A comparative summary of the advantages and disadvantages of the main drying methods is provided in Table 1.

d. Other drying techniques

In addition to the commonly used supercritical CO_2_ drying and freeze-drying methods for aerogel production, alternative drying techniques such as spray drying, microwave-assisted drying, and vacuum drying are employed for related porous materials or precursor powders. These methods typically offer shorter processing times or improved scalability; however, they are generally unsuitable for converting hydrogels into aerogels due to their tendency to reduce porosity and compromise structural integrity. The choice of drying method should therefore be carefully aligned with the target application, balancing factors such as cost, scalability, and desired material performance [12,24].

**Table 1:** Comparison of drying methods for aerogel production.

Drying Method	Advantages	Disadvantages
Air Drying	Simple and economical No specialized equipment required	High capillary tension may cause collapse Inconsistent porosity results
Freeze Drying	Preserves gel structure Produces highly porous aerogels No organic solvents required	Requires specialized equipment Long processing time High energy consumption Ice crystal formation
Supercritical Drying	Minimizes capillary forces, prevents collapse Produces aerogels with high porosity and low density Greater structural and mechanical stability	Complex and technical process Expensive and specialized equipment Requires solvent
Spray Drying	Fast and efficient for large volumes Allows control over product morphology Easy industrial scalability	Difficult to obtain porous structures Harsh thermal conditions
Microwave Drying	Fast and efficient in water removal	May cause hot spots and material decomposition Requires precise control of time and power

This table was created with information from [24,26,27].

## 6. Biodegradable Precursors for the Fabrication of Aerogels

Growing concerns about environmental sustainability and human health have intensified the pursuit of eco-friendly materials that can replace petroleum-derived synthetic polymers. Biodegradable polymers and other natural biomaterials sourced from agricultural and renewable resources offer a promising solution to mitigate the environmental impact associated with synthetic plastics [12,28].

Although traditional aerogels are primarily synthesized from inorganic or synthetic precursors such as silica, metal oxides, and polystyrene, increasing attention has been directed toward biopolymer-based aerogels. These biopolymer aerogels, particularly those derived from polysaccharides and proteins, are gaining traction in current food research due to their biodegradability, renewability, and functional performance [29,30]. This section reviews the main bio-based materials employed in bio-aerogel production.

### 6.1. Polysaccharide-Based Aerogels

Polysaccharides represent the most widely studied class of biopolymers for aerogel production due to their abundance, low cost, biocompatibility, and structural diversity. Hydrogels derived from natural polysaccharides such as starch, chitin, chitosan, agar, carrageenan, alginate, pectin, and cellulose have been commonly used as precursors for bio-aerogels [14,27,31].

Table 2 shows the structural and physical properties of polysaccharide-based aerogels from various studies. Due to their low density, surface area, and porosity, these aerogels have the potential to encapsulate and release bioactive compounds, which are essential for some types of active food packaging.

**Table 2:** Polysaccharide-based aerogels: structure and properties.

Polysaccharide	Properties	Dry Method	References
Corn starch (52.6% amylose)	Surface area: 1.3–1.7 m^2^/g; Pore volume: 0.0017–0.0054 cm^3^/g; Pore size: 14.9–15.1 nm; Porosity: 66.2–70.4%	Supercritical CO_2_ drying	[32]
Wheat starch	Surface area: 49.4–45.4 m^2^/g; Pore size: 0.09–0.27 cm^3^/g; Density: 0.03–0.05 g/cm^3^; Porosity: 91.3%	Supercritical CO_2_ drying	[33]
Alginate/pectin	Surface area: 16.76–21.27 m^2^/g; Pore size: 183–1081 nm; Density: 0.19–0.297 g/cm^3^; Porosity: 65.6–79%	Freeze-drying	[34,35]
Alginate/hyaluronic acid and Sodium alginate–grapefruit	Surface area: 446–611 m^2^/g; Density: 0.035–0.063 g/cm^3^; Porosity: 97–98% Thermal conductivity: 0.027–0.040 W · m^−1^ · K^−1^; Density: 0.030–0.042 g/cm^3^; Compressive strength: 317 kPa	Supercritical CO_2_ drying	[36,37]
Carrageenan (various types)	Surface area: 34–174 m^2^/g; Pore volume: 0.10–0.54 cm^3^/g; Pore size: 7.4–16.5 nm; Porosity: >94.3%	Supercritical CO_2_ drying	[38]
Konjac glucomannan/ soy protein	Density: 0.0201–0.0524 g/cm^3^; Porosity: 92.49–97.17%	Freeze-drying	[39]
Xanthan gum, gellan, and dextran	Lightweight, porous structures suitable for encapsulation and delivery	Supercritical CO_2_ drying	[26]
Chitosan	Surface area: 178 m^2^/g; Pore volume: 0.98 cm^3^/g; Porosity: ~96%; Density:0.034–0.063 g/cm^3^	Supercritical CO_2_ drying	[17]
Microcrystalline cellulose-based carbon	Surface area: 38 m^2^/g; Pore volume: 0.3–2.4 cm^3^/g; Pore size: 10–100 nm	Supercritical CO_2_ drying	[40]
Nanofibrillated cellulose	Pore size: surface area: 80–100 m^2^/g; nm; Density: 0.012–0.033 g/cm^3^; Porosity: 98–99%	Freeze-drying	[41]
k-Carrageenan	Density: 0.129–0.237 g/cm^3^; Porosity: 98–99%	Supercritical CO_2_ drying	[42]
Starch/cellulose	Pore size: 24.73–100 nm; Density: 0.012–0.033 g/cm^3^; Porosity: 64–87%	Freeze-drying	[43]

### 6.2. Protein-Based Aerogels

Vegetable and animal proteins are also prominent candidates in the development of bio-aerogels, though they are considerably less explored than polysaccharides. While traditionally utilized in drug delivery and biomedical scaffolds, their biocompatibility, structural versatility, and film-forming ability make them attractive for applications in active food packaging. Their lightweight, porous matrices can encapsulate natural preservatives or oxygen scavengers, enhancing food shelf life and sustainability. In the food sector, most research has focused on whey and egg white proteins as aerogel precursors [19,26].

Denatured whey proteins are known to undergo irreversible aggregation, forming interconnected gel networks suitable for aerogel formation [26]. However, a key limitation of whey protein aerogels is their poor mechanical strength. Even with increased initial protein concentrations, the resulting aerogels remained fragile and mechanically weak. Table 3 summarizes the structural characteristics, drying methods, and functional properties of protein-based aerogels reported in the recent literature to provide a clearer overview.

**Table 3:** Protein-based aerogels: structure and properties.

Protein	Properties	Dry Method	References
Whey protein isolate	Surface area: 354 m^2^/g, Pore volume: 1.55 cm^3^/g, Density: 0.28 g/cm^3^, Pore size: 79.1	Supercritical CO_2_ drying	[9,44]
Egg white protein	Surface area: 232 m^2^/g, Pore volume: 2.28 cm^3^/g, Density: 0.179 g/cm^3^, Pore size: 41.7 nm, Oil absorption: 0.74 g oil/g aerogel	Supercritical CO_2_ drying	[45]
Egg white protein isolate	Surface area: 154 m^2^/g, Pore volume: 0.33 cm^3^/g, Pore size: 7.1 nm	Supercritical CO_2_ drying	[46]
Egg white protein	Surface area: 390–422 m^2^/g, Pore volume: 1.27–1.69 cm^3^/g, Pore size: 9.2–14 nm	Supercritical CO_2_ drying	[19]
Soy protein	Surface area: 222–278 m^2^/g, Pore volume: 1.88–3.13 cm^3^/g, Density: 0.21 g/cm^3^, Pore size: 8–11 nm	Supercritical CO_2_ drying	[47]
Silk fibroin	Surface area: 424 m^2^/g, Pore size: 5–130 nm, Density: 0.19–0.25 g/cm^3^	Supercritical CO_2_ drying	[48]
Silk fibroin	Surface area: 260–308 m^2^/g, Pore size: 17 nm, Pore volume: 1.8–1.7 cm^3^/g	Supercritical CO_2_ drying	[49]
Plant-based isolates (pea, soy, chia seed, wheat, zein, lentil)	Protein-based aerogels with biocompatibility and porosity	Supercritical CO_2_ drying	[26,50,51,52]
Soy protein	Surface area: 384–478 m^2^/g, P: 17 nm, Pore volume: 0.12–0.15 cm^3^/g (micropore), 1.72–2.29 cm^3^/g (mesopore), 1.41–2.72 cm^3^/g (macropore), Density: 0.19–0.25 g/cm^3^	Supercritical CO_2_ drying	[24]

Table 3 shows the structural and physical properties of protein-based aerogels from various studies. Due to their low density, surface area, porosity, and oil absorption, these aerogels have the potential to become encapsulating or superabsorbent materials in active food packaging.

### 6.3. Hybrid Aerogels

The process of forming aerogels from proteins or polysaccharides yields bio-aerogels, which possess distinctive properties that are determined by the material from which they are derived. However, mixtures of proteins and polysaccharides have been demonstrated to improve their individual functional properties. This approach has driven the development of hybrid or composite bio-aerogels [29,30]. In this context, hybrid bio-aerogels can be classified into two broad categories:

i. Organic–Organic Bio-aerogels

These complexes are composed of multiple organic materials, resulting in various combinations, including protein–polysaccharide, protein–protein, and polysaccharide–polysaccharide. Notably, protein–polysaccharide systems exhibit distinct advantages, including enhanced water retention, improved texture, and increased stability. These properties are attributable to a variety of interactions between the biomolecules, including electrostatic, hydrophobic, hydrogen bonds and covalent interactions [53,54]. These interactions allow the modulation of both rheological properties and protein conformation. This phenomenon is attributed to the action of polysaccharides, which have been shown to reduce intermolecular distances and alter the protein microenvironment [53]. This alteration facilitates protein aggregation and cross-linking, resulting in gels with increased mechanical strength.

ii. Organic–Inorganic Bio-aerogels

These are obtained from an organic matrix that is functionalized with inorganic materials, such as nanoparticles, nanotubes, or nanofibers. This combination offers additional properties, including antimicrobial or antioxidant activity. It helps overcome the typical limitations of aerogels based on a single material, such as low mechanical strength and structural instability [54]. Table 4 shows a compendium of studies on different types of hybrid bio-aerogels and their potential food applications.

Furthermore, proteins are often incorporated into hybrid aerogels to improve structural integrity. For example, soy proteins help transition the morphology from fibrillar to continuous networks in cellulose protein aerogels [53], while zein has been used as a sacrificial porosity to introduce macroporosity in starch-based aerogels [14]. Moreover, blending whey protein with other biopolymers increased the viscosity of the precursor, but also made it more likely that air bubbles would become entrapped, creating structural defects and reducing homogeneity. In addition to offering protection, packaging materials must also fulfill other essential functions such as facilitating transportation and storage and conveying information to consumers [43]. The selection of appropriate packaging materials is guided not only by performance but also by factors such as cost-effectiveness, environmental impact, origin of raw materials, sustainability of the production process, and potential for recycling.

In recent years, the concept of food packaging has expanded beyond passive containment to include smart and active functionalities. Smart packaging systems can monitor product conditions and shelf life, while active packaging materials interact with the food or its environment to enhance preservation, often by absorbing or releasing functional compounds [1]. Within this context, bio-aerogels have emerged as promising candidates due to their unique physicochemical properties.

The properties of bio-aerogels enable their use not only as insulators and mechanical barriers but also as active packaging components capable of interacting with the packaged food [21,23]. The mechanical performance of these materials is closely linked to their internal structure; thus, reinforcements such as nanoparticles or natural fibers are often incorporated to enhance durability. These modifications contribute to more robust materials that are better able to protect food during transportation and storage.

**Table 4:** Hybrid bio-aerogels for active food packaging.

Matrix	Functional Material	Properties	Drying Method	Targeted Applications	References
Whey protein isolate (WPI)/Chitosan	Citric acid (CA), ε-polylysine hydrochloride (ε-PLH)	Superabsorbent (1486% water absorption); Antibacterial (≈80% against *S. aureus*, *E. coli*); Improves meat shelf-life (7 days)	Freeze-drying	Chicken meat preservation (absorbent pads)	[54]
Whey protein isolate (WPI)	Tannins	Reduced water absorption (219–559% vs. 4794% for pure WPI); Surface area: 216–353 m^2^/g	Supercritical CO_2_ drying	Food packaging (moisture-resistant)	[7]
WPI/Tannin	Bis(trimethylsilyl)amine (HMDS)	Hydrophobized (water absorption: 39–84%); Surface area: 87–242 m^2^/g	Supercritical CO_2_ drying	Food packaging (aqueous stability)	[7]
Dialdehyde nanocellulose (NCF)/Collagen	Sodium periodate (NaIO_4_)	High porosity (90–95%), Superabsorbent (>4000% water absorption); Low density: 0.025 g/cm^3^	Freeze-drying	Biological compatibility applications	[55]
Gelatin, Dialdehyde Starch, Bacterial Cellulose	Curcumin	Super absorbent (water: 30.86 g/g, oil: 27.67 g/g); Antibacterial (survival rate <45% for *E. coli*, *S. aureus*, *L. monocytogenes*); Resilience under 70% compression strain	Freeze-drying	Fresh pork preservation: extends shelf life to 12 days (absorbent pads)	[56]
Pectin/Alginate	Zinc oxide nanoparticles (ZnO)	Pore size: 0.18–0.54 μm, Antimicrobial activity against *S. aureus*, *E. coli*; Thermal stability; Water absorption (472–791%)	Supercritical CO_2_ drying	Antimicrobial food packaging	[57]
Whey Proteins	Spirulin (SP) cells	Low density: 0.23–0.29 g/cm^3^; High porosity; Firmness: 10–47.5 N, Absorption capacity (oil: 5.6 g/g, water: 5 g/g)	Supercritical CO_2_ drying	Food applications	[58]
Whey Proteins	Hydrophilic (alginate, agar) or hydrophobic (ethylcellulose) coatings	Low density: 0.28–0.35 g/cm^3^; Porosity: 74–79%; Firmness: 10–90 N; Absorption capacity (oil: 2–6.2 g/g, water: 6.5–8.5 g/g)	Supercritical CO_2_ drying	Active coatings/layers for food packaging and smart food ingredients	[9]
Starch/Cellulose	*Thymus daenensis* essential oil (TDEO)	Low density: 18.42–54.77 mg/cm^3^; Pore size: 24.73–95.5 μm; Antimicrobial activity against *E. coli O157:H7*, psychrophiles, and yeast-mold	Freeze-drying	Antimicrobial packaging for cheese	[43]
Chitosan	Copper nanoparticles (CuNPs) encapsulated in liposomes	Antimicrobial against Gram-positive and Gram-negative bacteria; Absorption capacity (oil: 17–25 g/g, water: 3–25 g/g); Density: 25–30 mg/cm^3^	Freeze-drying	Fresh pork preservation: extends shelf life to 14 days at 4 °C	[17]
Chitosan	Morillonite, clove essential oil, nanocellulose immobilized copper nanoparticles (CuNPs) fibers	Water absorption: ≈20%; Low density: 0.04–0.06 g/cm^3^; Porosity: 54.4–77.4%; Antimicrobial activity against *E. coli*, *S. aureus* and mold; Resilience under 30% compression strain	Freeze-drying	Active packaging and buffers for food (fruits and vegetables): protects blueberries from damage during transport and extends the storage by 3 days at 20 °C and 85% humidity	[28]
Poly(vinyl alcohol) (PVA)	Silica aerogel (SA)	Thermal conductivity: 0.068 W m^−1^·K^−1^); Tensile strength: 18.05–42.32 MPa; Water vapor transmission rate: 1.28–1.76 g m^−2^ d^−1^; Thermal stability	Not described	Multilayer packaging system for temperature-sensitive foodstuff packaging applications: chocolate	[10]
Galactoglucomannan (GGM), Cellulose Nanofibrils	Sunflower oil (SFO)	Density: ≈0.02 g/cm^3^; Surface area: 2–4 m^2^/g; Hexanal release for at least three weeks: 7–23 µmol/g	Freeze-drying	Food packaging materials with a system for in situ production and release hexanal: tests of blueberries and cherry tomatoes	[59]
Carboxymethyl Nanocellulose (CMC)/Chitosan/glycerol	Silver nanoparticles (AgNPs)	Cushioning coefficient: 5.04; Compression resilience (>90%); Antibacterial against *E. coli*, *S. aureus*; Biodegradation of >70% within 14 days; Swelling rate: 116.67%	Freeze-drying	Cushioning and antibacterial packaging for the storage and transportation of fruits and vegetables	[60]
Alginate	Oxidized nanocellulose	Porosity: 81–97.4%; Water absorption: 793–1468%; Water retention: 221.1–846.7%; Thermal stability	Freeze-drying	Food packaging for temperature-sensitive foods	[61]

Table 4 shows the structural and physicochemical properties of hybrid aerogels from various studies. Incorporating inorganic materials, bioactive agents, and other functional materials optimizes the properties of bio-aerogels, resulting in greater stability, both physicochemical and mechanical. Additionally, functionalization provides antimicrobial, antioxidant, thermal insulation, barrier, and superabsorbent properties. These materials have proven effective in active food packaging, extending shelf life and protecting different types of food.

## 7. Applications of Bio-Aerogels in Food Packaging

### 7.1. Application in Fruit and Vegetable Packaging

Fruits and vegetables are highly perishable and prone to degradation due to oxidative processes and microbial activity during postharvest handling. Such spoilage leads to considerable losses in sensory and nutritional attributes. The use of bio-aerogels in packaging offers dual benefits: mechanical protection and active preservation, which limits exposure to spoilage-promoting factors. This is often achieved through chemical functionalization of the bio-aerogel or the controlled release of bioactive compounds, thereby extending product shelf life [59,62].

An example is the development of an active bio-aerogel system, formed from a polysaccharide matrix of galactoglucomannan (GGM) reinforced with anionic cellulose nanofibrils (CNF) and incorporated with sunflower oil as a substrate for the in situ generation and release of hexanal, a volatile compound known to suppress ethylene production and inhibit microbial growth, delaying ripening and spoilage. In comparative evaluations, packaging containers embedded with this bio-aerogel demonstrated significantly reduced mold formation in blueberries and enhanced firmness retention in tomatoes, outperforming conventional packaging solutions [59].

Similarly, Franco et al. [21] developed a bio-aerogel matrix composed of corn starch and calcium alginate, incorporating quercetin, a natural antioxidant and antimicrobial agent, via supercritical CO_2_ adsorption. This active bio-aerogel layer gradually releases quercetin, providing extended protection against spoilage and enhancing the overall preservation capacity of the packaging system.

### 7.2. Application in Fresh Meat Packaging

Fresh meat is particularly susceptible to microbial spoilage due to its high-water activity and nutrient-rich composition. Spoilage not only leads to economic loss but also poses significant food safety concerns. In this context, bio-aerogels offer promising solutions owing to their superabsorbent capacity, which allows them to retain fluids such as exudates or blood, thereby reducing microbial proliferation and preserving product quality [54].

Recent studies have shown that aerogels can be tailored to exhibit multifunctional properties, including moisture absorption and the controlled release of antimicrobial agents [15,29]. For example, antimicrobial bio-aerogels have been designed from dialdehyde starch, chitosan, and copper nanoparticles. These bio-aerogels successfully preserved fresh pork for up to 14 days at 4 °C without visible signs of spoilage. Their effectiveness is attributed to the synergistic action of antimicrobial components and the absorbent matrix [17].

Another approach was presented by [14,59], who designed corn starch- and starch/cellulose-based aerogels loaded with bioactive compounds. These bio-aerogels not only exhibited strong water absorption capacity but also acted as carriers for antioxidants, enhancing the overall protective function of the packaging. Additionally, Zhou et al. [60] further advanced the field by fabricating a multifunctional bio-aerogel based on nanocellulose through coaxial 3D printing, yielding a core–shell architecture. The outer shell consisted of carboxymethylated nanocellulose, acrylamide derivatives, and glycerol, while the core encapsulated chitosan and silver nanoparticles. Bio-based aerogel demonstrated significant antimicrobial activity against *Escherichia coli*, as reported by [17], combined with high buffering capacity and mechanical resilience.

### 7.3. Other Applications of Bio-Aerogels in Food Packaging

Due to their versatility in modulating properties, bio-aerogels have been explored for various solutions in active food packaging. For example, they have been proposed as oxygen and moisture scavengers for processed snacks and dry foods to minimize oxidation and extend shelf life [1]. Similarly, studies have developed aerogels from Arundo donax biomass waste that reduce lipid oxidation and preserve the red color of beef during refrigeration [63]. Another study designed whey protein aerogels combined with chitosan and loaded with ε-polylysine to significantly reduce microbial growth in chicken meat [54]. These results demonstrate the potential of bio-aerogels to prevent oxidation and improve the quality of various types of foods.

Regarding their absorption properties, wheat starch aerogels with polyethylene glycol (PEO) were developed in [64], which demonstrated high water absorption capacity and favorable moisture control in food packaging. Similarly, corn starch aerogels with glycerol were developed to absorb moisture in spinach packaging, extending its shelf life to 10 days under refrigeration [65]. Furthermore, to improve the structural integrity and thermal protection of food products, polyvinyl alcohol (PVA) films reinforced with silica aerogel were developed to provide thermal insulation and protect chocolates from high temperatures [10]. Additionally, 3D printing was used to produce nanocellulose and chitosan aerogels containing silver nanoparticles in [62], which offer cushioning and antimicrobial properties, making them a promising alternative for transporting food susceptible to physical damage.

## 8. Conclusions and Prospects

The integration of aerogels into food packaging systems must comply with stringent safety and regulatory frameworks to ensure consumer protection. According to Regulation (EC) No. 1935/2004, materials intended to come into contact with food must be inert and must not release substances that could endanger human health, alter the organoleptic properties of food, or change its composition. In the case of active and intelligent packaging materials, Regulation (EC) No. 450/2009 allows certain interactions, such as the absorption of oxygen or moisture, the controlled release of preservatives, or the monitoring of food quality. However, these non-inert materials must undergo rigorous safety assessments, typically overseen by the European Food Safety Authority (EFSA). One approach to mitigating potential risks associated with aerogels is the application of a protective barrier coating, which can limit direct contact with food and reduce the likelihood of undesirable interactions [53].

A promising strategy for the sustainable production of aerogels is the direct conversion of plant residues to obtain precursors, which simplifies processing and valorizes agricultural waste [2,12]. Increasingly, research has focused on using low-value, renewable biomass as a feedstock for aerogel fabrication in packaging applications. While such raw materials are less cost-effective, the processing methods, particularly those involving high-pressure drying techniques like supercritical CO_2_, can be technically demanding and economically burdensome. Nevertheless, innovations aimed at reducing solvent consumption and optimizing drying procedures may significantly lower production costs and enhance the scalability of aerogel manufacturing [11].

Two key technical challenges must be addressed to advance the practical application of aerogels in food packaging. First, the poor visual transparency of bio-based aerogels remains a limitation, as consumers generally prefer packaging that enables product visibility. However, fully transparent packaging may be less effective at protecting food from spoilage factors such as light, oxygen, and heat. Consequently, the challenge is to appropriately reduce packaging transparency for the benefit of extended food storage [14]. The introduction of new materials such as aerogels in food packaging implies that consumer acceptance and sensory evaluation are crucial to ensure that the package design meets consumer preferences [8]. The slow depressurization process during supercritical drying helps maintain the aerogel network morphology, decreasing the formation of fissures that cause opacity [23]. Recent advances have shown that highly transparent aerogels can be produced using oxidized cellulose, thus expanding their commercial potential and aesthetic appeal [61]. To summarize, while consumers desire packaging transparency to visualize the product and its freshness indicators, manufacturers must balance transparency with the need to provide effective protection against spoilage factors to ensure food safety and quality. Second, the recyclability and reusability of aerogel-based packaging remain an open question, as they depend on the material composition and whether the aerogel is integrated into multilayer or composite systems. This, in turn, affects the viability of conventional recycling processes and end-of-life management strategies.

Ultimately, the broader adoption of aerogels in food packaging urges the establishment of clear regulatory guidelines and standardized testing protocols to confirm their safety, including the development of validated analytical methods to assess potential interactions with food and biological systems. Addressing these scientific and regulatory challenges is critical for enabling the responsible and widespread use of aerogels in next-generation sustainable packaging solutions.

## Data Availability

Data availability is not applicable to this article, as no new data were created in this study.

## References

[1] Dis Huang K., Wang Y. (2025). Advances in bio-based smart food packaging for enhanced food safety. Trends Food Sci. Technol..

[2] Taherimehr M., Yousefnia Pasha H., Tabatabaeekoloor R., Pesaranhajiabbas E. (2021). Trends and challenges of biopolymer-based nanocomposites in food packaging. Compr. Rev. Food Sci. Food Saf..

[3] Grand View Research, Inc Biopolymers Market Size, Share and Growth Report, 2030. https://www.grandviewresearch.com/industry-analysis/biopolymers-market-report.

[4] Ncube L.K., Ude A.U., Ogunmuyiwa E.N., Zulkifli R., Beas I.N. (2020). Environmental impact of food packaging materials: A review of contemporary development from conventional plastics to polylactic acid based materials. Materials.

[5] Siddaway A.P., Wood A.M., Hedges L.V. (2019). How to do a systematic review: A best practice guide for conducting and reporting narrative reviews, meta-analyses, and meta-syntheses. Annu. Rev. Psychol..

[6] Kistler S.S. (1931). Coherent expanded aerogels and jellies. Nature.

[7] Effraimopoulou E., Kalmár J., Paul G., Marchese L., Ioannou D., Paraskevopoulou P., Gurikov P. (2024). Whey protein isolate-based aerogels with improved hydration properties for food packaging applications. ACS Appl. Nano Mater..

[8] Leite A.C., Pereira R.N., Rodrigues R.M. (2025). Protein aerogels as food-grade delivery systems—A comprehensive review. Food Hydrocoll..

[9] De Berardinis L., Plazzotta S., Magnan M., Manzocco L. (2024). Hydrophilic or hydrophobic coating of whey protein aerogels obtained by supercritical-CO_2_-drying: Effect on physical properties, moisture adsorption and interaction with water and oil in food systems. Innov. Food Sci. Emerg. Technol..

[10] Chen C., Ding R., Yang S., Wang J., Chen W., Zong L., Xie J. (2020). Development of thermal insulation packaging film based on poly(vinyl alcohol) incorporated with silica aerogel for food packaging application. LWT—Food Sci. Technol..

[11] Dhua S., Gupta A.K., Mishra P. (2022). Aerogel: Functional emerging material for potential application in food: A review. Food Bioprocess Technol..

[12] Wei G., Zhang J., Usuelli M., Zhang X., Liu B., Mezzenga R. (2022). Biomass vs inorganic and plastic-based aerogels: Structural design, functional tailoring, resource-efficient applications and sustainability analysis. Prog. Mater. Sci..

[13] Santos P.D., Viganó J., De Figueiredo Furtado G., Cunha R.L., Hubinger M.D., Rezende C.A., Martínez J. (2020). Production of resveratrol-loaded alginate aerogel: Characterization, mathematical modeling, and study of impregnation. J. Supercrit. Fluids.

[14] Zhao D., Zhang X., Zhang Y., Xu E., Yan S., Xu H., Li M. (2024). Recent advances in the fabrication, characterization and application of starch-based materials for active food packaging: Hydrogels and aerogels. Sustain. Food Technol..

[15] Kazemi-Taskooh Z., Varidi M. (2021). Designation and characterization of cold-set whey protein-gellan gum hydrogel for iron entrapment. Food Hydrocoll..

[16] Vrabič-Brodnjak U. (2024). Hybrid Materials of Bio-Based Aerogels for Sustainable Packaging Solutions. Gels.

[17] Chen L., Niu X., Fan X., Liu Y., Yang J., Xu X., Zhou G., Zhu B., Ullah N., Feng X. (2022). Highly absorbent antibacterial chitosan-based aerogels for shelf-life extension of fresh pork. Food Control.

[18] Falua K.J., Babaei-Ghazvini A., Acharya B. (2024). Comparative study of the structure and mechanical properties of starch aerogels fabricated from air-classified and isolated pulse starches. Int. J. Biol. Macromol..

[19] Kleemann C., Schuster R., Rosenecker E., Selmer I., Smirnova I., Kulozik U. (2020). In-vitro-digestion and swelling kinetics of whey protein, egg white protein and sodium caseinate aerogels. Food Hydrocoll..

[20] Alavi F., Ciftci O.N. (2023). Effect of starch type and chitosan supplementation on physicochemical properties, morphology, and oil structuring capacity of composite starch bioaerogels. Food Hydrocoll..

[21] Franco P., Aliakbarian B., Perego P., Reverchon E., De Marco I. (2018). Supercritical adsorption of quercetin on aerogels for active packaging applications. Ind. Eng. Chem. Res..

[22] Klost M., Keil C., Gurikov P. (2024). Dried Porous Biomaterials from Mealworm Protein Gels: Proof of Concept and Impact of Drying Method on Structural Properties and Zinc Retention. Gels.

[23] Basak S., Singhal R.S. (2023). The potential of supercritical drying as a “Green” method for the production of food-grade bioaerogels: A comprehensive critical review. Food Hydrocoll..

[24] Amaral-Labat G., Grishechko L., Szczurek A., Fierro V., Pizzi A., Kuznetsov B., Celzard A. (2012). Highly mesoporous organic aerogels derived from soy and tannin. Green Chem..

[25] Liu H., Xing F., Yu P., Zhe M., Shakya S., Liu M., Ritz U. (2024). Multifunctional aerogel: A unique and advanced biomaterial for tissue regeneration and repair. Mater. Des..

[26] Selvasekaran P., Chidambaram R. (2021). Food-grade aerogels obtained from polysaccharides, proteins, and seed mucilages: Role as a carrier matrix of functional food ingredients. Trends Food Sci. Technol..

[27] Baudron V., Gurikov P., Smirnova I., Whitehouse S. (2019). Porous starch materials via supercritical- and freeze-drying. Gels.

[28] Wang X., Guo J., Zhou H., Hou Y., Jin P., Zheng Y., Wu Z. (2025). Chitosan-based aerogel food active packaging with integrated preservation and buffering functions. J. Adv. Res..

[29] Chel A., Hazarika M. (2020). Application of aerogels in the packaging of fresh meat: A review. Packag. Technol. Sci..

[30] Nita L.E., Ghilan A., Rusu A.G., Neamtu I., Chiriac A.P. (2020). New trends in bio-based aerogels. Pharmaceutics.

[31] Wang Y., Su Y., Wang W., Fang Y., Riffat S.B., Jiang F. (2019). The advances of polysaccharide-based aerogels: Preparation and potential application. Carbohydr. Polym..

[32] Goimil L., Braga M.E.M., Dias A.M.A., Gómez-Amoza J.L., Concheiro A., Alvarez-Lorenzo C., de Sousa H.C., García-González C.A. (2017). Supercritical processing of starch aerogels and aerogel-loaded poly(ε-caprolactone) scaffolds for sustained release of ketoprofen for bone regeneration. J. CO2 Util..

[33] Ubeyitogullari A., Ciftci O.N. (2016). Formation of nanoporous aerogels from wheat starch. Carbohydr. Polym..

[34] Xu J., Zhang Y., Wang J., Zhang H., Zhang W., Liu J., Zhang Y., Liu X. (2024). Advanced alginate-based nanofiber aerogels. Compos. Sci. Technol..

[35] Chen K., Zhang H. (2019). Alginate/pectin aerogel microspheres for controlled release of proanthocyanidins. Int. J. Biol. Macromol..

[36] Athamneh T., Amin A., Benke E., Ambrus R., Leopold C.S., Gurikov P., Smirnova I. (2019). Alginate and hybrid alginate–hyaluronic acid aerogel microspheres as potential carrier for pulmonary drug delivery. J. Supercrit. Fluids.

[37] Jing N., Feng Y., Ge H., Tang Q., Xie Y., Li S. (2025). Sodium alginate–grapefruit peel aerogels with outstanding thermal conductivity, excellent compressive strength, and low density. Mater. Today Commun..

[38] Alnaief M., Obaidat R., Mashaqbeh H. (2018). Effect of processing parameters on preparation of carrageenan aerogel microparticles. Carbohydr. Polym..

[39] Ran X., Yang H. (2022). Promoted strain-hardening and crystallinity of a soy protein-konjac glucomannan complex gel by konjac glucomannan. Food Hydrocoll..

[40] Alatalo S., Pileidis F.D., Mäkilä E., Sevilla M., Salonen J.J., Sillanpää M. (2015). Versatile cellulose based carbon aerogel for the removal of both cationic and anionic metal contaminants from water. ACS Appl. Mater. Interfaces.

[41] Jiménez-Saelices C., Seantier B., Cathala B., Grohens Y. (2017). Spray freeze-dried nanofibrillated cellulose aerogels with thermal superinsulating properties. Carbohydr. Polym..

[42] Manzocco L., Valoppi F., Calligaris S., Andreatta F., Spilimbergo S., Nicoli M. (2017). Exploitation of κ-carrageenan aerogels as template for edible oleogel preparation. Food Hydrocoll..

[43] Mirmoeini S.S., Hosseini S.H., Lotfi Javid A., Esmaeili Koutamehr M., Sharafi H., Molaei R., Moradi M. (2023). Essential oil-loaded starch/cellulose aerogel: Preparation, characterization and application in cheese packaging. Int. J. Biol. Macromol..

[44] Ahmadi M., Madadlou A., Sabouri A.A. (2015). Whey protein aerogel as blended with cellulose crystalline particles or loaded with fish oil. Food Chem..

[45] Selmer I., Karnetzke J., Kleemann C., Lehtonen M., Mikkonen K.S., Kulozik U., Smirnova I. (2019). Encapsulation of fish oil in protein aerogel micro-particles. J. Food Eng..

[46] Selmer I., Kleemann C., Kulozik U., Heinrich S., Smirnova I. (2015). Development of egg white protein aerogels as new matrix material for microencapsulation in food. J. Supercrit. Fluids.

[47] Kaur S., Singh S., Saini R., Sharma S., Kaur A., Kaur A., Kaur M., Kaur P., Kaur J., Kaur G. (2025). Transforming soy proteins into nanoporous aerogels using supercritical CO_2_ drying. J. Food Sci..

[48] Marín M., Mallepally R., McHugh M.A. (2014). Silk fibroin aerogels for drug delivery applications. J. Supercrit. Fluids.

[49] Mallepally R.R., Marin M.A., Surampudi V., Subia B., Rao R.R. (2015). Silk fibroin aerogels: Potential scaffolds for tissue engineering applications. Biomed. Mater..

[50] Chen Q., Guan J., Wang Z., Wang Y., Wang X., Chen Z. (2024). Improving the Gelation Properties of Pea Protein Isolates Using Psyllium Husk Powder: Insight into the Underlying Mechanism. Foods.

[51] Mekala S., Saldaña M.D.A. (2023). Lentil protein concentrate–pectin gels dried with supercritical CO_2_: Influence of protein–polysaccharide interactions on the characteristics of aerogels. J. Supercrit. Fluids.

[52] Yang C., Li A., Guo T., Cheng J., Liu Z., Hu H., Wang J. (2024). Novel organic-inorganic composite pea protein silica food-grade aerogel materials: Fabrication, mechanisms, high oil-holding property and curcumin delivery capacity. Int. J. Biol. Macromol..

[53] Zhu Y., Li H., Peng C., Ma J., Huang S., Wang R., Chen J. (2023). Application of protein/polysaccharide aerogels in drug delivery system: A review. Int. J. Biol. Macromol..

[54] Zhou X., Guo X., Chai Y., Li X., Chen L., Feng X. (2024). Superabsorbent whey protein isolates/chitosan-based antibacterial aerogels: Preparation, characterization and application in chicken meat preservation. Int. J. Biol. Macromol..

[55] Lu T., Li Q., Chen W., Yu H. (2014). Composite aerogels based on dialdehyde nanocellulose and collagen for potential applications as wound dressing and tissue engineering scaffold. Compos. Sci. Technol..

[56] Wang F., Xu Z., Chen L., Qiao Z., Hu Y., Fan X., Liu Y., Kang Z., Huang F., Han M. (2024). Super absorbent resilience antibacterial aerogel with curcumin for fresh pork preservation. Food Control.

[57] Mottola S., Viscusi G., Oliva G., Vigliotta G., Cardea S., Gorrasi G., De Marco I. (2025). Pectin/alginate aerogel containing ZnO produced from beetroot extract mediated green synthesis for potential applications in food packaging. J. CO2 Util..

[58] De Berardinis L., Plazzotta S., Magnan M., Manzocco L. (2024). Hybrid aerogels of spirulina and whey proteins as novel cellular solids. LWT.

[59] Lehtonen M., Kekäläinen S., Nikkilä I., Kilpeläinen P., Tenkanen M., Mikkonen K.S. (2020). Active food packaging through controlled in situ production and release of hexanal. Food Chem. X.

[60] Zhou W., Fang J., Tang S., Wu Z., Wang X. (2021). 3D-printed nanocellulose-based cushioning-antibacterial dual-function food packaging aerogel. Molecules.

[61] Lin N., Bruzzese C., Dufresne A. (2012). TEMPO-oxidized nanocellulose participating as crosslinking aid for alginate-based sponges. ACS Appl. Mater. Interfaces.

[62] Ezati P., Khan A., Priyadarshi R., Bhattacharya T., Tammina S.K., Rhim J.W. (2023). Biopolymer-based UV protection functional films for food packaging. Food Hydrocoll..

[63] Fontes-Candia C., Erboz E., Martínez-Abad A., López-Rubio A., Martínez-Sanz M. (2019). Superabsorbent food packaging bioactive cellulose-based aerogels from Arundo donax waste biomass. Food Hydrocoll..

[64] Da Silva F.T., de Oliveira J.P., Fonseca L.M., Bruni G.P., Da Rosa Zavareze E., Dias A.R.G. (2020). Physically cross-linked aerogels based on germinated and non-germinated wheat starch and PEO for application as water absorbers for food packaging. Int. J. Biol. Macromol..

[65] Dhua S., Mishra P. (2023). Development of highly reusable, mechanically stable corn starch-based aerogel using glycerol for potential application in the storage of fresh spinach leaves. Int. J. Biol. Macromol..

